# Acute Transverse Myelitis Following COVID-19 Infection: A Rare Case From Saudi Arabia

**DOI:** 10.7759/cureus.17658

**Published:** 2021-09-02

**Authors:** Mohamad Bakir, Fatimah Rebh

**Affiliations:** 1 Medicine and Surgery, Alfaisal University, College of Medicine, Riyadh, SAU; 2 Department of Internal Medicine, Section of Infectious Diseases, Prince Mohammed Bin Abdulaziz Hospital, Riyadh, SAU

**Keywords:** covid-19, sars-cov-2, transverse myelitis, spinal cord injury, complication, case report, kingdom of saudi arabia (ksa)

## Abstract

Respiratory viral illnesses can lead to a wide variety of neurological complications. However, only a few cases of acute transverse myelitis (ATM) following severe acute respiratory syndrome coronavirus 2 (SARS-CoV-2) infection have been reported in the literature. Here, we report a case of ATM following SARS-CoV-2 infection in a 57-year-old male patient. The patient presented to the emergency room with lower abdominal pain, urinary retention, bilateral lower limbs weakness, and allodynia for the last four days. One week earlier, he had experienced fever, cough, and shortness of breath. On physical examination, he was vitally stable with sensory loss from the nipples down to the lower limbs bilaterally. His nasopharyngeal polymerase chain reaction for SARS-CoV-2 was positive. MRI of the spine showed an abnormal cord signal extending from the level of the D2 vertebra down to the conus medullaris. The main differential diagnosis was transverse myelitis, and the patient was started on pulse steroids for seven days. After the therapy, the condition of the patient improved with the restoration of power and sensory sensation in his lower limbs. A new MRI of the whole spine one month later showed normal morphology and signal intensity without any abnormal enhancement. The patient was discharged home with almost complete resolution of his symptoms for later follow-up in the clinic.

## Introduction

Coronaviruses have been linked to illnesses such as the common cold, Middle East respiratory syndrome, and severe acute respiratory syndrome (SARS). A new coronavirus, known as the severe acute respiratory syndrome coronavirus 2 (SARS-CoV-2), was discovered in China in 2019. The World Health Organization named the disease coronavirus disease 2019 (COVID-19) on February 11, 2020. COVID-19 is used to prescribe a mixture of clinical symptoms secondary to SARS-CoV-2. Respiratory viral illnesses can lead to a wide variety of neurological complications. However, there are only a few reported cases in the literature about acute transverse myelitis (ATM) after SARS-CoV-2 infection [1]. Transverse myelitis is an inflammation of one or more spinal cord levels with no concomitant compressive lesions. It is divided into acute and subacute inflammatory myelopathy; the disease can be disabling resulting in motor weakness, sensory deficits, and autonomic dysfunction [2]. ATM is a rare neurological disorder in adults, with an incidence of 1.34-4.6 per million people per year, with an average age of onset of 35-40 years [3]. Here, we present the case of a 57-year-old male patient who developed ATM secondary to COVID-19 infection.

## Case presentation

A 57-year-old male patient, a known case of diabetes mellitus type 2, presented to the emergency department with lower abdominal pain, urinary retention, lower limb weakness, and allodynia for four days before admission. One week earlier, he had experienced fever, cough, and shortness of breath, and he reported a history of contact with a COVID-19-positive patient. The patient denied any history of back trauma or receiving a new vaccination or medications. On physical examination, he was conscious, alert, and oriented to time, place, and person. In addition, the patient’s vital signs were stable. He had a sensory loss from the nipples down to the lower limbs bilaterally, along with a painful burning sensation in the same area with no skin rash or vesicular lesions noticed. There was no history of joint pain, gastrointestinal symptoms, and fecal or urinary incontinence. He had negative meningeal signs, and his cranial nerves were intact. The power in the upper and lower limbs was 5/5 and 2/5, respectively. He had decreased patellar reflex, and plantar reflex could not be assessed due to severe pain expressed by the patient in that area. The pain score from the nipples below was 5 out of 10 without touch and 10 out of 10 with touch. His Glasgow Coma Scale score was 15/15, and his urine dipstick was normal. His laboratory workup at the time of admission is presented in Table 1.

**Table 1:** Lab values at the time of admission. CSF: cerebrospinal fluid; PCR: polymerase chain reaction; SARS-CoV-2: severe acute respiratory syndrome coronavirus 2

Laboratory test	Findings
Urine analysis	Negative
Urine culture	Negative
Blood random glucose	306 mg/dL (Normal range: 74-106 mg/dL)
C-reactive protein	0.91 mg/dL (Normal range: 0-1 mg/dL)
Cytoplasmic antineutrophil cytoplasmic antibodies	Negative
Antinuclear antibody	Negative
Anti-double-stranded DNA	Negative
Perinuclear antineutrophil cytoplasmic antibodies	Negative
Anti-Smith antibodies	Negative
Nasopharyngeal PCR for SARS-CoV-2	Positive
CSF cell count analysis	50 cells with 69% lymphocytes (Normal range: 0-5 cells/µL)
CSF glucose level	4.92 mmol/L (Normal range: >60% of the serum glucose)
CSF protein level	192 mg/dL (Normal range: <45 mg/dL)
CSF culture	Negative
CSF PCR for cytomegalovirus	Negative
CSF PCR for herpes simplex virus	Negative
CSF PCR for tuberculosis	Negative

**Figure 1:**
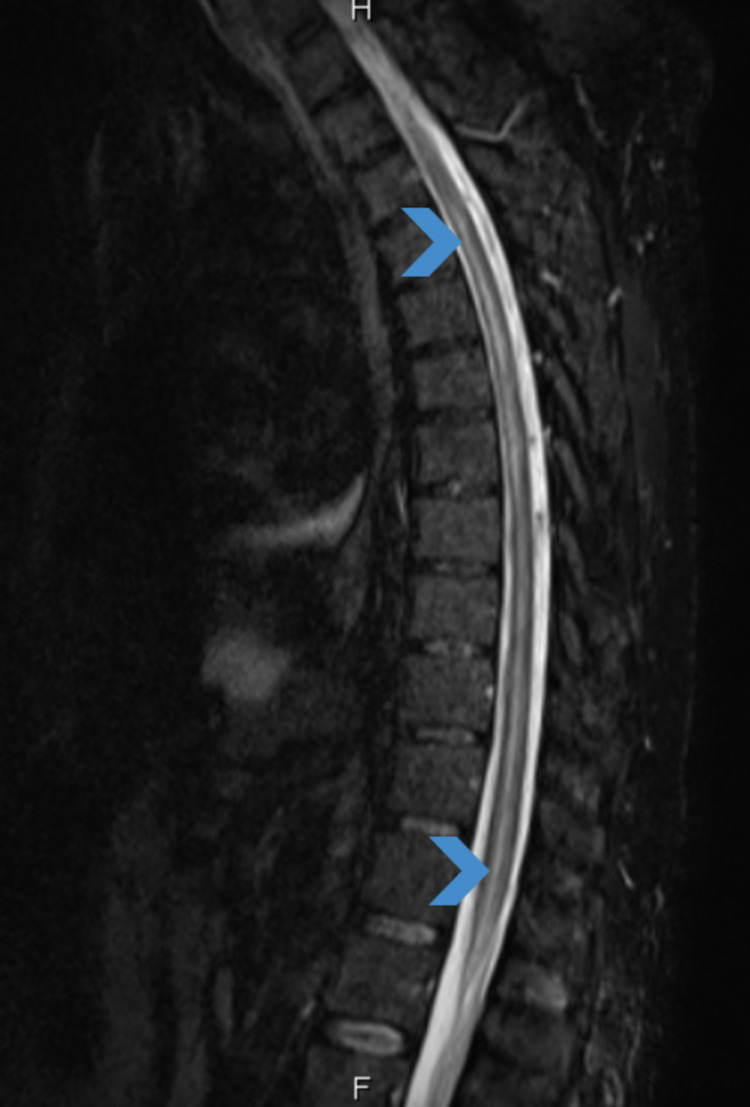
Sagittal short tau inversion recovery MRI of the dorsal spine pretreatment. The MRI demonstrates long segment intramedullary abnormal high signal intensity. MRI: magnetic resonance imaging

**Figure 2:**
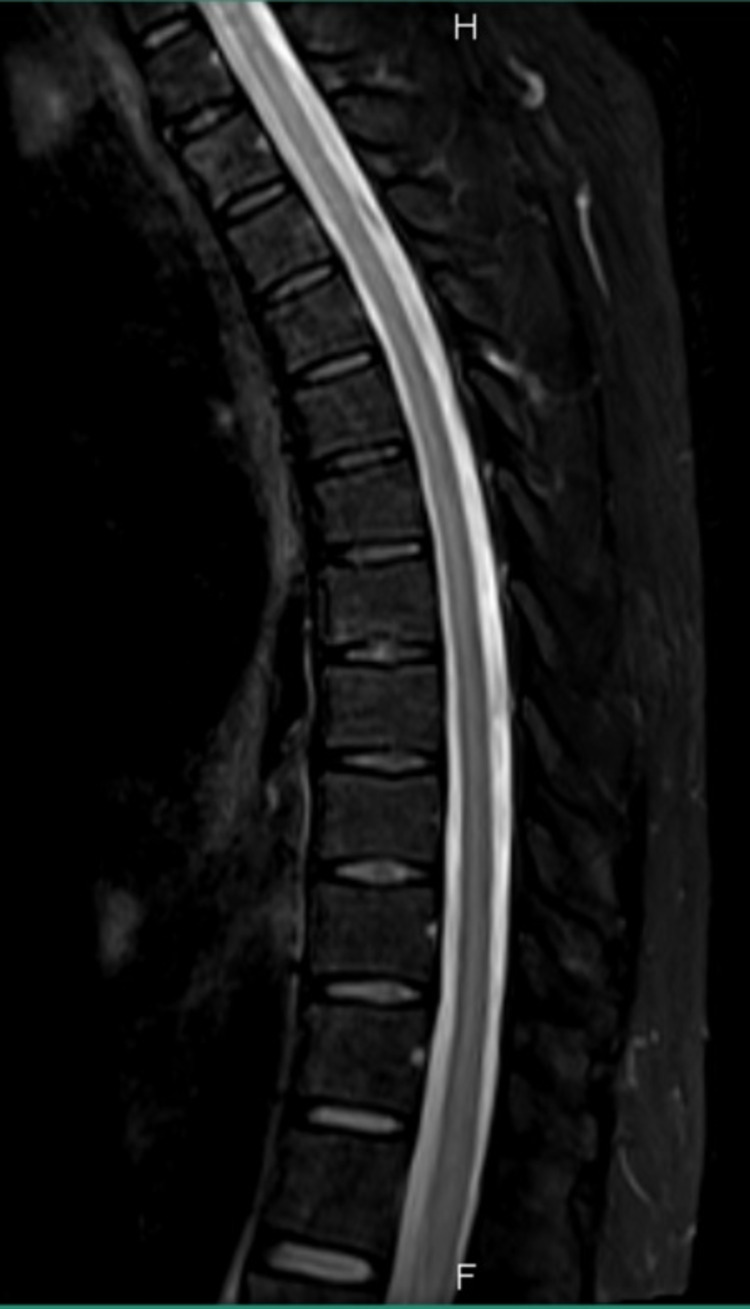
Sagittal short tau inversion recovery MRI of the dorsal spine posttreatment. The MRI demonstrates normal morphology and resolution of the signal intensity noted earlier. MRI: magnetic resonance imaging

Complete blood count (CBC), vitamin B12, folate, and thyroid-stimulating hormone levels were within the normal range. Brain CT was normal. Therefore, an MRI spine was requested which showed an abnormal cord signal extending from the level of the D2 vertebra down to the conus medullaris, involving mainly the gray matter in the proximal thoracic cord and most of the axial cross-section of the conus medullaris and distal part of the cord (Figure 1). The involvement of the gray matter was suggestive of ischemia, and the main differential diagnosis was transverse myelitis. The patient was started on 1 g intravenous (IV) pulse steroid (methylprednisolone) in the morning over three hours for seven days along with physiotherapy and strict control of his blood sugar levels. Seven days later, his condition improved, and the power in his lower limbs improved to 4/5 proximally and distally. Furthermore, the patient started to feel numbness and tingling sensation in his lower limbs. Consequently, the patient continued physiotherapy in addition to bladder training for the following month. As a part of a follow-up plan, a new MRI of the whole spine was performed to compare with the previous study obtained one month ago. On the new MRI, complete disappearance of the signal intensity was noted in the thoracic region, along with normal morphology and signal intensity without any abnormal enhancement (Figure 2). The patient was discharged home with almost complete resolution of his symptoms for later follow-up in the clinic.

## Discussion

Transverse myelitis is an inflammation on both sides of one or more spinal cord sections. However, there is not always complete anatomical damage across the cord, but rather localized inflammation that might result in asymmetric spinal cord dysfunction below the location of the injury. According to one study, the cervical region is the most affected part in idiopathic acute transverse myelitis (60%), followed by the thoracic region (33%) [4]. Although the causes of ATM are extensive, they can be divided into compressive and noncompressive myelopathies. Noncompressive causes include infectious, post-infectious, post-vaccination, ischemic, autoimmune, radiation, paraneoplastic, and idiopathic causes [5]. The onset of transverse myelitis can be acute within hours to days and is associated with a poorer prognosis. On the other hand, the subacute onset of transverse myelitis lasting from one to four weeks is associated with good to decent outcomes [6,7]. It is crucial to diagnose ATM promptly to avoid the long-term consequences that can leave two-thirds of the patients with moderate-to-severe permanent disability [5,8,9]. MRI is the gold standard in diagnosing transverse myelitis because it can not only visualize the lesion but also rule out other treatable causes. Patients with transverse myelitis caused by multiple sclerosis, infectious diseases, or idiopathic reasons are younger, whereas those with transverse myelitis caused by delayed radiation effects or spinal cord infarcts are older [10]. Recovery may or may not be complete depending on the clinical presentation. However, one-third of the patients have a good recovery, another one-third have a moderate recovery, while one-third of the patients fail to recover or pass away [11]. COVID-19 can involve the central nervous system either hematogenously or through neuronal antegrade or retrograde spread via the peripheral nerves [12]. There have been reported cases of ATM post-COVID-19 infection where the patient had a negative cerebrospinal fluid polymerase chain reaction test for the COVID-19 virus [1]. An explanation for the post-SARS-CoV-2 infection ATM is the immunological mimicry process, where the immune system that targets the infectious agent starts attacking the central and peripheral nervous systems, a process that results in neuronal death and spinal tract damage [13]. ATM can be a challenging diagnosis as in most cases it is a diagnosis of exclusion where most of the other etiologies need to be ruled out before we conclude ATM. The efficacy of high-dose IV methylprednisolone, antivirals, and intravenous immunoglobulin in ATM have been reported in the literature. However, the treatment should be tailored to each patient [14]. In this paper, we have reported a challenging case of ATM post-SARS-CoV-2 infection. Because ATM can render the patient in severe neurological sequelae if not treated early, sharing the experience is crucial.

## Conclusions

Transverse myelitis is an inflammatory condition that affects one or more segments of the spinal cord. Pain, paralysis, muscle weakness, sensory problems, and bowel and bladder dysfunction can result from this condition. In very rare instances, transverse myelitis can be a complication of COVID-19 infection. Early diagnosis by MRI and management with pulse steroids (methylprednisolone) is a crucial and lifesaving intervention to prevent long-term complications. Hence, transverse myelitis should be considered as a differential diagnosis for a COVID-19-positive patient who presents with a neurological deficit.
